# Venomics of the Arabian saw-scaled viper (*Echis coloratus*) through transcriptome-guided proteomics and in vitro functional profiling

**DOI:** 10.1371/journal.pntd.0013439

**Published:** 2025-08-14

**Authors:** Ignazio Avella, Lennart Schulte, Maik Damm, Lilien Uhrig, Alfredo Cabrera-Orefice, Johanna Eichberg, Kornelia Hardes, Sabine Hurka, Thomas Lindner, Andreas Vilcinskas, Tim Lüddecke

**Affiliations:** 1 Animal Venomics Lab, Fraunhofer Institute for Molecular Biology and Applied Ecology (IME), Giessen, Germany; 2 Institute for Insect Biotechnology, Justus Liebig University, Giessen, Germany; 3 LOEWE Centre for Translational Biodiversity Genomics (LOEWE-TBG), Frankfurt am Main, Germany; 4 Institute of Biochemistry, Medical Faculty, Justus Liebig University, Giessen, Germany; 5 Branch of Bioresources, Fraunhofer Institute for Molecular Biology and Applied Ecology (IME), Giessen, Germany; 6 BMBF Junior Research Group in Infection Research “ASCRIBE”, Giessen, Germany; 7 BMBF Junior Research Group in Bioeconomy (BioKreativ) “SymBioÖkonomie”, Giessen, Germany; 8 Institute for Zoology and Evolutionary Biology, University of Regensburg, Regensburg, Germany; 9 Museum für Naturkunde – Leibniz Institute for Evolution and Biodiversity Science, Berlin, Germany; Fundação de Medicina Tropical Doutor Heitor Vieira Dourado: Fundacao de Medicina Tropical Doutor Heitor Vieira Dourado, BRAZIL

## Abstract

The Arabian saw-scaled viper (*Echis coloratus*) is among the snakes of highest medical relevance in the Middle East and North Africa. However, to date, its venom has been investigated in a very limited number of studies, and much remains unknown regarding its compositional and functional properties. By integrating proteotranscriptomics with bioactivity profiling, we present a comprehensive transcriptome-level catalogue of *E. coloratus* venom components and their associated biological activities. Our analysis identified 183 venom components belonging to 17 distinct protein families. Relative toxin abundances revealed that 92% of the venom proteome is composed of C-type lectin and C-type lectin-related protein (CTL), L-amino acid oxidase (LAAO), phospholipase A_2_ (PLA_2_), snake venom serine protease (SVSP), and snake venom metalloproteinase (SVMP), with CTL and PLA_2_ alone accounting for 73% of the total composition. Bioassays targeting key aspects of viperid envenomation demonstrated potent protease and PLA_2_ activity in a concentration-dependent manner. In contrast, Factor Xa-like, plasmin-like, and haemolytic activities were negligible. Marked cytotoxicity was observed at the highest concentration tested (i.e., 25 μg/ml) in the mammalian cell lines MDCK II and Calu-3, whereas cytotoxic effects were minimal at lower concentrations. These findings highlight the complexity and potency of *E. coloratus* venom, and provide a valuable foundation for improving our understanding of envenomation caused by this species.

## Author summary

The Arabian saw-scaled viper (*Echis coloratus*) is a venomous snake of high medical relevance found in the Middle East and North Africa. However, the compositional and functional properties of its venom remain poorly understood. In this study, we investigated both the composition and effects of this species’ venom by combining protein analysis with biological assays that measure venom activity. We identified approximately 200 venom-related proteins across 17 different families, with two families accounting for nearly three-quarters of the total venom. We observed strong activity from enzymatic compounds typically associated with disruption of the blood system and tissue damage. Interestingly, effects on blood clotting factors and red blood cells were very weak. We also saw that, at high concentrations, the venom could kill a high percentage of mammalian cells in culture. Our findings provide a clearer picture of the complexity and potency of Arabian saw-scaled viper venom, which may help to improve medical treatment for people affected by bites from this species.

## Introduction

Snakebite envenoming is a neglected tropical disease that was officially recognised by the World Health Organization in 2017 [1]. It affects millions of people worldwide each year, with the most severe impacts observed in rural communities across the Global South [2–4]. Due to the remarkable diversity of snake venom components, along with variations in their relative abundances and activities, the clinical manifestations of envenoming are highly variable [5]. Envenomation symptoms range from localised and mild (e.g., swelling, moderate pain) to systemic and potentially life-threatening (e.g., neuromuscular paralysis, haemorrhage) [3,6]. The wide spectrum of clinical symptoms associated with snakebite envenoming can be broadly categorised into three main groups, based on the predominant pathophysiological mechanisms of the venom: i) neurotoxic, impairing nervous system function; ii) cytotoxic, causing cellular damage or death, and/or inhibiting cell growth; and iii) haemotoxic, affecting the blood system and disrupting haemostasis [6,7]. In light of this variety, characterising components and activities of venoms from medically relevant snakes is crucial for the development of effective therapies and tools to manage and treat snakebite.

The burden of snakebite envenoming is the highest in Sub-Saharan Africa, Asia, and Latin America [3,8], but it also represents a serious public health concern in the Middle East and North Africa (MENA) region [9–11]. This vast area harbours several species of venomous snakes, including some highly medically relevant ones, such as vipers of the genera *Bitis*, *Echis*, and *Macrovipera*, as well as cobras (genus *Naja*) [10–13]. These snakes pose a considerable threat to the estimated 800 million people residing in the MENA region, particularly those leading a rural lifestyle [14]. However, assessing the burden of snakebite in MENA remains challenging, due to the general lack of reliable epidemiological data and reports [10,15,16], and much remains to be understood on the toxin compositions and properties of the venoms of the many medically relevant snake species from this region.

Snakes of the genus *Echis*, commonly known as “saw-scaled vipers” or “carpet vipers”, are widely distributed across central and northern Africa, the Middle East, and extend as far as India and Sri Lanka [17,18]. This genus comprises approximately a dozen species, recognised among the Old World vipers (family Viperidae, subfamily Viperinae) of the highest clinical importance, and accounting for a substantial proportion of the global snakebite burden [11,19]. Their venoms follow the typical composition of viperine venoms, and predominantly comprise snake venom metalloproteinase (SVMP), snake venom serine protease (SVSP), phospholipase A_2_ (PLA_2_), C-type lectin and C-type lectin-related protein (CTL), and disintegrin (DI) [20]. Viperid venoms mainly cause haemotoxic and cytotoxic effects [3,6], and envenomation by saw-scaled vipers aligns with this pattern, typically resulting in haemostatic alterations such as extensive haemorrhage and coagulopathy [15,21]. Due to the severe clinical effects caused by their bites, snakes of the genus *Echis* are often regarded as leading contributors to snakebite-induced human morbidity and mortality within their geographical range [15,22].

**Fig 1:**
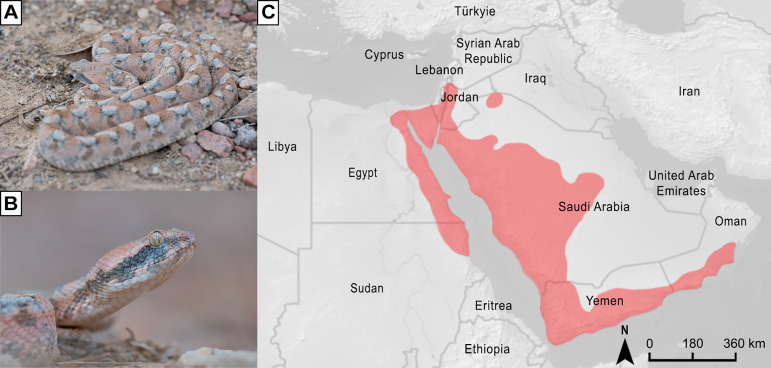
Appearance and distribution of the Arabian saw-scaled viper (*Echis coloratus*). (A) Full-body photograph and (B) close-up of adult *E. coloratus*. (C) Geographic distribution of the species, based on data from the WHO Snakebite Information and Data Platform [11]. The map’s base layer was sourced from Natural Earth (https://www.naturalearthdata.com/downloads/50m-raster-data), which is in the public domain (http://www.naturalearthdata.com/about/terms-of-use). Photo credits: Thor Håkonsen.

Over the years, numerous studies have investigated composition and activities of saw-scaled viper venoms, not only from a toxicological and medical perspective, but also from an evolutionary point of view [23–25]. However, certain species within the genus *Echis* have thus far received little attention in snake venom research. One such species is the Arabian saw-scaled viper, *Echis coloratus*. Also known as the “painted saw-scaled viper”, “Burton’s carpet viper”, and by various other common names, this relatively slender and variably coloured venomous snake reaches a maximum total length of approximately 80 cm [26]. It occurs from north-eastern Sudan and eastern Egypt to the southern coast of Oman, including Israel, Jordan, Palestine, Saudi Arabia, Syria, and Yemen [11] (Fig 1).

Bites by *E. coloratus* can result in local symptoms such as extensive swelling, haemorrhagic blisters, and necrosis at the bite site [27]. Systemic symptoms are predominantly haemotoxic, with documented cases of severe haemorrhagic shock, thrombocytopenia, and coagulopathy [28–30], and complications such as renal toxicity and thrombotic microangiopathy have also been reported following envenomation by this species [15,31]. Despite the medical relevance of *E. coloratus* and the severity of the symptoms it can induce, the composition and activities of its venom have yet to be thoroughly investigated, leaving a substantial gap in our understanding of the toxinology of the genus *Echis*.

In this study, we utilise the venom gland transcriptome of the Arabian saw-scaled viper [32], combined with an in-depth shotgun proteomics analysis, to provide a thorough characterisation of *E. coloratus* venom composition. Furthermore, we investigate its functional implications by assessing its protease, PLA_2_, Factor Xa-like, thrombin-like, and plasmin-like activities, alongside its haemolytic and cytotoxic properties. Our study presents a comprehensive insight into the compositional diversity of *E. coloratus* venom and offers an extensive analysis of its biological activities, establishing a robust foundation for understanding and managing envenomation caused by this species.

## Methods

### Venom samples

Pooled, lyophilised *E. coloratus* venom was commercially sourced (Ophiotox, Germany), and comprised of venom samples obtained from four adult (SVL > 600 mm) captive individuals (two males and two females) originating from the Sinai Peninsula (Egypt). The venom was stored at −20°C until analysis.

### Profiling the composition of *E. coloratus* venom

Following the procedure previously applied by our group [33–35], we analysed the protein composition of *E. coloratus* venom using three different approaches: i) mass spectrometry (MS) shotgun proteomics, ii) reverse-phase high-performance liquid chromatography (RP-HPLC), and iii) sodium dodecyl sulphate polyacrylamide gel electrophoresis (SDS-PAGE).

#### Shotgun proteomics.

Given that proteins and peptides constitute approximately 90% of the dry weight of snake venom, as clearly indicated in the existing literature [36,37], we did not determine the protein concentration of *E. coloratus* venom prior to analysis. The lyophilised venom was dissolved in 6 M guanidine hydrochloride (GdnHCl), 100 mM Tris/HCl at pH 8.5 to ensure complete denaturation. The final concentration was 1.7 µg venom/µl. Aliquots containing 100 µg of venom were transferred to Protein LoBind tubes (Eppendorf) and supplemented with 10 mM dithiothreitol (DTT) for disulphide bond reduction, and incubated at 37°C for 30 minutes. Free thiols were subsequently alkylated using 40 mM chloroacetamide, with incubation at room temperature (22°C) in the dark for 30 minutes. Subsequently, a Trypsin/LysC mix (Promega) was added at a 1:50 protease-to-venom ratio, and samples were incubated at 37°C with shaking at 500 rpm for one hour. To reduce the concentration of GdnHCl (< 1 M) and reactivate trypsin, samples were diluted 1:7 with 50 mM Tris/HCl at pH 8.0. Proteolysis was then continued overnight at 37°C with shaking at 500 rpm. After 15 h, digestion was terminated by the addition of 1.5% trifluoroacetic acid (TFA). Peptides were subsequently desalted and purified using C18-Chromabond columns (Macherey-Nagel). The final eluate was dried in a vacuum concentrator (Eppendorf) at 45°C for three hours, then resuspended in 20 µl of 5% acetonitrile (ACN) and 0.15% formic acid (FA) with vortex mixing. The peptide solution was transferred to 96-well PCR plates, sonicated in a water bath for five minutes, and then prepared for LC-MS/MS analysis. Prior to mass spectrometry analysis, chromatographic separation of peptides was conducted using a Thermo Fisher Scientific UltiMate 3000 RSLCnano system. From the prepared peptide mixture, 5 µl was loaded onto a PepMap Neo Trap column (Thermo Fisher Scientific) for concentration and desalting. Peptides were then separated using a 50 cm µPAC column (PharmaFluidics, Thermo Fisher Scientific) interfaced with a TriVersa NanoMate (Advion) for chip-based nano-electrospray ionisation. The analytical column was maintained at 35°C throughout the runs. Peptide elution was achieved using a linear gradient of ACN acidified with 0.1% FA, increasing from 5% to 35% over 90 minutes, followed by a further increase to 85% ACN over 10 minutes at a flow rate of 700 nl/min. The column was subsequently washed for eight minutes with 85% ACN containing 0.1% FA and re-equilibrated with 5% ACN and 0.1% FA for at least 10 minutes. Mass spectrometry analysis was performed using an Orbitrap Eclipse Tribrid MS (Thermo Fisher Scientific) operating in positive ion mode. Electrospray ionisation was facilitated by the NanoMate system, applying a voltage of 1.7 kV, with the ion source temperature set to 250°C. Data acquisition was conducted in data-dependent (DDA) mode.

For protein annotation, PEAKS Studio 12.0 (build 20240725; Bioinformatics Solutions Inc.) was employed with the following parameters: Parent Mass Error Tolerance set to 15.0 ppm, Fragment Mass Error Tolerance at 0.5 Da, Precursor Mass Search Type as monoisotopic, Enzyme specified as Trypsin, with a maximum of three missed cleavages. A semi-specific digestion mode was applied, and the peptide length range was defined as 5–45 residues. Post-translational modifications (PTMs) included carbamidomethylation (+57.02) as a fixed modification, while variable modifications comprised acetylation (K; + 42.01), oxidation (MHW; + 15.99), pyro-glu from E (−18.01) and pyro-glu from Q (−17.03), methylation (KR; + 14.02), phosphorylation (STY; 79.97), Hexose (NSY; + 162.05), HexNAcylation (N; + 203.08) and sodium adduct (+21.98) with a limit of four variable PTMs per peptide.

The database search was conducted against the *E. coloratus* venom gland transcriptome data published by Hargreaves et al. [32]. Particularly, we used the publicly available RNA sequencing datasets ERR216311, ERR216312, SRR1287707, and SRR1287715, which were derived from venom glands collected at different time points after venom extraction [32]. The data underwent quality assessment with FastQC 0.12.1 (https://www.bioinformatics.babraham.ac.uk/projects/fastqc). Quality trimming and adapter removal were performed with cutadapt 5.0 (minlength: 25; quality: 28) [38]. Subsequent assemblies were generated using Trinity 2.15.2 (max_read_cov: 50; min_contig_length: 30) [39] and two runs of rnaSPAdes 4.0.0 (kmers: 21,33,55) [40], with one run utilizing corrected reads from Rcorrector 1.0.7 [41]. The three assemblies were merged, and duplicate contigs were eliminated using fastanrdb from the exonerate suite 2.4.0 [42]. Open reading frames (ORFs) were identified using TransDecoder 5.7.1 (minimum length: 10) [39], followed by signal peptide prediction using SignalP 6h (mode: slow_sequential; organism: eukarya) [43]. All ORFs lacking signal peptide and stop codon were excluded, providing the database for proteome analysis. The putative peptides were annotated using DIAMOND blastp 2.1.10 (sensitivity mode: ultra; max target seqs: 0/all) [44] against the UniProtKB 2024_06-based [45] databases Swiss-Prot, TrEMBL (Serpentes, taxonomy ID 8570), VenomZone (https://venomzone.expasy.org), and Tox-Prot [46] (all accessed on 26 January 2025). For each DIAMOND hit, query and subject coverage, as well as sequence similarity, were computed using the BLOSUM62 matrix [47] with BioPython 1.85 [48]. The results were arranged by similarity, query, and subject coverage in descending order for each venom candidate, with the top DIAMOND hit selected for further analysis. Functional annotation was performed using InterProScan 5.72-103.0 [49], assigning putative venom components to their respective venom families. Data transformation was performed with SAMtools 1.21 [50]. The relative abundances of the components identified in the analysed *E. coloratus* venom, based on the Normalized Spectral Abundance Factor (NSAF) of each protein, were calculated as described by Avella et al. [35].

Details of all identified venom components and their calculated relative abundances are provided in S1–S3 Tables. Mass spectrometry proteomics data have been deposited in the Zenodo repository (https://zenodo.org) under the project identifier 15127165 [51].

#### Reverse-phase high-performance liquid chromatography (RP-HPLC).

For the RP-HPLC analysis, 200 μg of lyophilised venom were diluted in 20 µl of double-distilled water (ddH_2_O) with 5% (v/v) acetonitrile (ACN), containing 1% (v/v) formic acid (FA), and centrifuged at 12,000 × g for 10 minutes. The venom was profiled with an Agilent 1100 HPLC system (Agilent Technologies, Waldbronn, Germany) equipped with a Supelco Discovery BIO wide pore C18-3 column (4.6 × 150 mm, 3 µm particle size), operating at 40°C at a flowrate of 1 mL/min. The following linear gradient steps were applied with solvent A (ultrapure water with 0.1% (v/v) FA) and solvent B (acetonitrile (ACN) with 0.1% (v/v) FA): 0–5 min (5% B, constant), 5–65 min (5% to 45% B), 65–75 min (45% to 70% B), 75–80 min (95% B, constant), followed by a 10-minute re-equilibration at 5% B. Wavelength was observed at λ = 214 nm and at λ = 280 nm through a diode array detector (DAD), using a reference wavelength of λ = 360 nm. Process control and data acquisition were performed with OpenLAB CDS ChemStation (version 2.15.26, Agilent). A blank run (5% (v/v) ACN with 1% (v/v) FA, centrifuged at 12,000 × g for 10 minutes) was performed under the same conditions and subtracted from the venom profile to clear the chromatogram from background noise.

#### Gel electrophoresis (SDS-PAGE).

For one-dimensional SDS-PAGE profiling, 5 μg of lyophilised *E. coloratus* venom was dissolved in ddH_2_O, and combined with either a non-reducing or a reducing Laemmli buffer, the latter containing 5% (v/v) 2-mercaptoethanol. The sample was then incubated at 95°C for 5 min before being loaded onto 4–20% Mini-PROTEAN TGX Precast Protein Gels (Bio-Rad). Electrophoresis was performed for 60 minutes at 120 V using a Mini-PROTEAN Tetra Vertical Electrophoresis Cell (Bio-Rad). A molecular weight marker (Precision Plus Protein All Blue Standard 10–250 kDa, Bio-Rad) was included for reference. Proteins were visualised using ROTI Blue quick stain (Carl Roth), and subsequently destained with a solution containing 10% acetic acid and 50% methanol. Digital images of the gels were captured using a Molecular Imager Gel Doc XR+ System with Image Lab Software 6.1.0 (Bio-Rad).

### Biological activity assays

We performed a range of plate-based biological assays to evaluate the protease, PLA_2_, Factor Xa-like, thrombin-like, plasmin-like, as well as the haemolytic and cytotoxic activities of *Echis coloratus* venom. The procedures, as outlined by Avella et al. [35], are briefly described below. Venom aliquots were diluted five-fold (50, 25, 12.5, 6.25, and 3.125 µg/ml) in the respective assay buffer. For incubation and signal measurements, a BioTek Eon microplate reader with Gen v2.09 software was used. For each assay, the results were normalised by first subtracting the values of the negative controls (corresponding to 0%) and then dividing by the difference between the positive control (corresponding to 100%) and the negative control, according to the following formula:


$$Relative\;activity\;(\%)=\frac{\overset{―}{x}\;sample\;-\;\overset{―}{x}\;negative\;control}{\overset{―}{x}\;positive\;control\;-\;\overset{―}{x}\;negative\;control}$$

where x̅ indicates the mean of the measured signals at specific wavelengths. Assays were conducted in triplicate, and results were expressed as the mean ± standard deviation. The amounts of venom used in the assays were quantified based on their dry weight.

#### Protease activity.

Protease activity was assessed employing a Pierce Fluorescent Protease Assay Kit (23266, Thermo Fisher Scientific) according to the manufacturer’s guidelines. Briefly, each venom concentration was tested against TPCK trypsin at final concentration of 2 µg/ml in Tris-buffered saline (TBS), used as positive control, and TBS, used as negative control. In a white, flat-bottom 96-well plate, a mixture containing 100 μl of 5µg/ml fluorescein thiocarbamoyl-casein derivatives (FTC-casein) in TBS, and 100 μl of either venom dilution or control was prepared. The plate was incubated at room temperature for 55 min, protected from light. Fluorescence was measured every 5 min at a gain of 75 with excitation set to λ = 485 nm and emission set to λ = 538 nm.

#### Phospholipase A_2_ activity.

Phospholipase A_2_ (PLA_2_) activity was assessed using an EnzChek Phospholipase A_2_ Assay Kit (E10217, Invitrogen) following the manufacturer’s guidelines. The positive control was prepared by diluting the 500 Units/ml bee venom’s PLA_2_ 1× in PLA_2_ reaction buffer. The negative control consisted of 1 × PLA_2_ reaction buffer. For each venom dilution and both controls, 50 μl were transferred into a black, flat-bottom 96-well plate, and 50 μl of the substrate-liposome mix were added to each well to start the reaction. The plate was incubated at room temperature for 55 min, protected from light. Fluorescence was measured every 5 min, at a gain of 50 with excitation set to λ = 470 nm and emission set to λ = 515 nm.

#### Factor Xa-like activity.

Factor Xa (FXa)-like activity was evaluated employing the Factor Xa Activity Fluorometric Assay Kit (MAK238-1KT, Sigma-Aldrich) following the manufacturer’s guidelines. For each measurement, 50 µl of assay substrate were added to a white, flat-bottom 96-well plate and kept shielded from light. Venom dilutions were added to the substrate in volumes of 50 µl. A final concentration of 0.4 µg/ml FXa Enzyme Standard was used as positive control, and 10% ddH_2_O in assay buffer as negative control. The plate was incubated at 37°C for 55 min, protected from light. FXa-like activity was detected at an excitation wavelength of λ = 350 nm and emission measured at λ = 450 nm.

#### Thrombin-like activity.

Thrombin-like activity was assessed using the Thrombin Activity Assay Kit (Fluorometric) (ab197006, Abcam) according to the manufacturer’s guidelines. For each measurement, 50 µl of assay substrate were placed into a black, flat-bottom 96-well plate with a transparent base and shielded from light. To start the reaction, 50 µl of venom dilutions were added. A final concentration of 0.3 µg/ml Thrombin Enzyme Standard and 10 (v/v) ddH_2_O in assay buffer served as the positive and the negative control, respectively. The plate was incubated at 37°C for 55 min, protected from light. Activity was measured with excitation set at λ = 350 nm and fluorescence emission detected at λ = 450 nm.

#### Plasmin-like activity.

Plasmin-like activity was assessed using the Plasmin Activity Assay Kit (Colorimetric) (ab273301, Abcam) following the manufacturer’s manual. For each measurement, 50 µl of assay substrate was dispensed into a clear, flat-bottom 96-well plate. The assay was started by adding 50 µl of venom dilutions. A final concentration of 0.1% (v/v) Plasmin Enzyme Standard in assay buffer as the positive control, and 10% (v/v) ddH_2_O in assay buffer as the negative control. The plate was incubated at 37°C for 55 min. Absorbance to determine activity was measured at λ = 405.

#### Haemolytic activity.

Haemolytic activity was assessed using an adapted version of the protocol described by Sæbø et al. [52]. Briefly, erythrocytes from defibrinated horse blood (0.3 ml, SR0050D, Thermo Fisher) were purified and diluted to a final concentration of 1% (w/v) in Dulbecco’s phosphate-buffered saline (DPBS; 0.2 g/l KCl, 0.2 g/l KH_2_PO_4_, 8.0 g/l NaCl, 2.1716 g/l Na_2_HPO_4_, pH 7.4) as previously described [35]. For each measurement, 50 µl of the 1% erythrocyte suspension was aliquoted into a transparent, V-bottom 96-well plate. In order to start the assay, 50 µl of venom dilutions were added to the diluted erythrocytes. A final concentration of 1% (v/v) aqueous Triton X-100 solution in DPBS was used as the positive control, and ddH_2_O (10% (v/v) in DPBS) as the negative control. The plate was incubated at 130 rpm for 55 min at 37°C on a Multitron shaker (Infors HT). Following incubation, the plate was centrifuged at 804 × g for 5 minutes at 4°C, and 50 µl of supernatant from each well was transferred to a transparent, flat-bottomed 96-well plate. Haemolytic activity was measured at an absorbance of λ = 405 nm.

#### Cytotoxic activity.

The cytotoxicity of *E. coloratus* venom was assessed on Madin-Darby canine kidney II (MDCK II) cells and the human epithelial cell line Calu-3 derived from lung adenocarcinoma (Merck, SCC438), following procedures previously applied by our group [33,53,54]. Cells were cultured in Dulbecco’s Modified Eagle’s Medium (DMEM) GlutaMAX, supplemented with 10% foetal calf serum (FCS) and 1% penicillin/streptomycin, and incubated at 37°C in a humidified atmosphere containing 5% CO_2_. Cells were seeded into 96-well plates and allowed to grow to confluence. Venom was prepared by dissolving it in water, while ionomycin (7.74 mg/ml stock solution, Cayman Chemicals) was dissolved in DMSO. The cells were treated with venom (25, 2.5, or 0.25 µg/ml), ddH_2_O (negative control), or ionomycin (100 µM, positive control), and incubated at 37°C in 5% CO_2_. After 48 hours, cell viability was determined using the CellTiter-Glo Luminescent Cell Viability Assay (Promega), following the manufacturer’s guidelines. Luminescence was measured in black 96-well plates using a Synergy H4 microplate reader (H4MLFPTAD, BioTek).

## Results

### Compositional profile of *E. coloratus* venom

**Fig 2:**
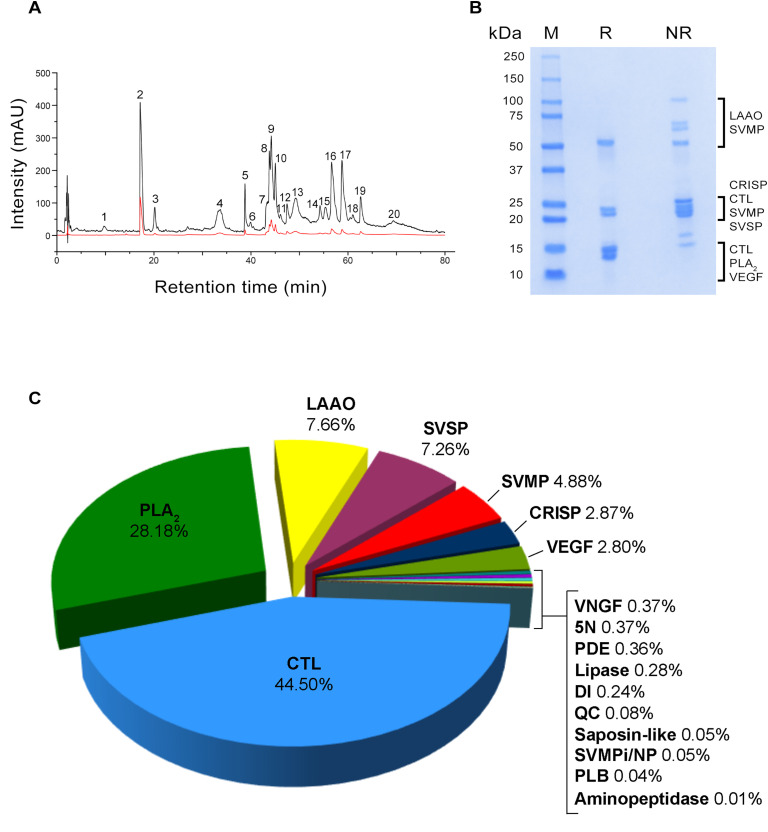
Characterisation of *E. coloratus* venom. (A) RP-HPLC profile at 214 nm (black) and 280 nm (red), (B) one-dimensional SDS-PAGE profiles, and (C) relative abundances of the venom components based on NSAFs. In the SDS-PAGE profiles, venom toxin families were tentatively assigned based on the molecular masses estimated from SDS-PAGE analysis of reduced samples reported by Casewell et al. [54]. Abbreviations: M, marker; R, reduced; NR, non-reduced. Toxin family key: 5N, 5′-nucleotidase; CTL, C-type lectin and C-type lectin-related protein; CRISP, cysteine-rich secretory protein; DI, disintegrin; PDE, phosphodiesterase; PLA_2_, phospholipase A_2_; PLB, phospholipase B; QC, glutaminyl-peptide cyclotransferase; LAAO, L-amino-acid oxidase; SVMP, snake venom metalloproteinase; SVMPi/NP, snake venom metalloproteinase inhibitors/natriuretic peptides; SVSP, snake venom serine protease; VEGF, venom endothelial growth factor; VNGF, venom nerve growth factor.

We identified 20 main peaks in the RP-HPLC profile of *E. coloratus* venom, the majority of which appeared within a retention time (RT) range of 30–70 minutes, alongside several minor peaks (Fig 2A). The most prominent peak was observed at a RT of approximately 17 minutes. One-dimensional SDS-PAGE analysis was performed under both reducing and non-reducing conditions (Fig 2B), and showed most protein bands within range of 13–60 kDa.

Following this initial profiling, a previously sequenced, high-quality venom gland transcriptome for *E. coloratus* [32] was utilised as a species-specific reference database for venom characterisation via bottom-up shotgun proteomics. This approach enabled the detection of 301 proteins within the venom sample (S1 Table). After applying rigorous quality control measures (i.e., DIAMOND protein searches, InterProScan, manual comparative alignment), 183 venom-related components were confidently identified, spanning 17 protein families: 5′-nucleotidase (5N), aminopeptidase, C-type lectin and C-type lectin-related protein (CTL), cysteine-rich secretory protein (CRISP), disintegrin (DI), phosphodiesterase (PDE), phospholipase A_2_ (PLA_2_), phospholipase B (PLB), glutaminyl-peptide cyclotransferase (QC), L-amino-acid oxidase (LAAO), lipase, saposin-like proteins, snake venom metalloproteinase (SVMP), SVMP inhibitors and natriuretic peptides (SVMPi/NP), snake venom serine protease (SVSP), venom endothelial growth factor (VEGF), and venom nerve growth factor (VNGF) (S2 and S3 Tables). The CTL family exhibited the greatest diversity, with 62 entries, followed by SVSP with 29 entries. The SVMP family was represented by 22 entries, distributed across three subfamilies: 19 in subfamily P-III, two in subfamily P-I, and one in subfamily P-II. Furthermore, 16 entries were retrieved for PLA_2_, and 10 for LAAO. Fewer than nine entries were attributed to each of the other identified toxin families, with VNGF being the least represented, having only a single entry (S2 Table).

Shotgun proteomics using in-solution trypsin digestion enabled us to detect a broad range of toxins within the analysed *E. coloratus* venom, including those present at low abundance. This was crucial for cataloguing the species’ complete toxin profile within a proteo-transcriptomic approach, using a species-specific database (i.e., venom gland transcriptome). To gain quantitative insights into the venom composition of the Arabian saw-scaled viper, the normalised spectral abundance factors (NSAFs) were calculated to estimate relative toxin abundance [55]. The most abundant toxin families were CTL (45%), PLA_2_ (28%), LAAO (8%), SVSP (7%), and SVMP (5%), accounting for 92% of the total venom composition. The remaining 8% comprised additional protein families, including CRISP (3%), VEGF (3%), 5N (0.4%), VNGF (0.4%), PDE (0.4%), lipase (0.3%), and DI (0.2%). Aminopeptidase, PLB, QC, SVMPi/NP and saposin-like proteins each contributed less than 0.1% of the venom proteome. Further details on the components identified in the analysed *E. coloratus* venom and their relative abundances is presented in Fig 2C and S2 and S3 Tables.

### Biological activity of *E. coloratus* venom

**Fig 3:**
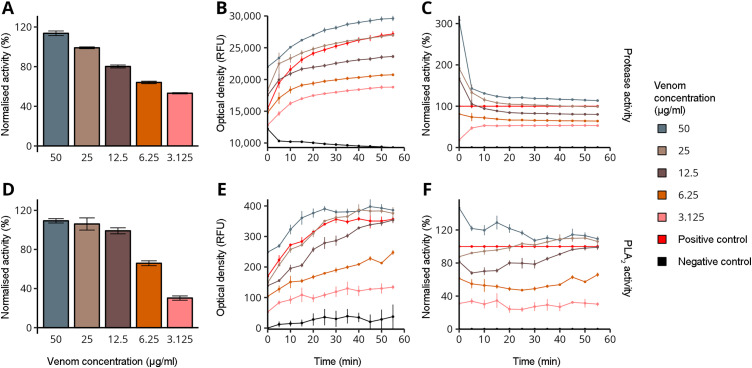
Protease and PLA_2_ activity of *E. coloratus* venom. The graphs show the results at the final time point, the kinetic profile, and the normalized kinetic profile for protease activity (panels A, B, C) and PLA_2_ activity (panels D, E, F). Panels B and E show the optical density measured at 600 nm and expressed in Relative Fluorescence Units (RFU). The error bars represent standard deviation. For the protease activity assay, TPCK trypsin was applied as positive control, and TBS as negative control. For the PLA_2_ activity assay, bee venom PLA_2_ diluted in 1 × Reaction Buffer to a working solution of 10 U/ml PLA_2_ was applied as positive control, and 1 × Reaction Buffer as negative control.

Given that SVMP, SVSP, and PLA_2_ are among the predominant enzymatic components of *Echis* venoms [20], we investigated the contribution of these toxins to envenomation by *E. coloratus*. To this end, we conducted assays to evaluate the protease and PLA_2_ activities of this species’ venom. We detected remarkably high protease activity, ranging from 53% (3.125 µg/ml) to over 100% (50 µg/ml), with a concentration-dependent trend (Fig 3A). The kinetic profile further revealed that venom concentrations of 50 µg/ml exhibited protease activity exceeding the positive control throughout the entire 55-minute incubation period (Fig 3B and 3C). A similar concentration-dependent relationship was observed for PLA_2_ activity, which ranged from 30% (3.125 µg/ml) to 99% (12.5 µg/ml), and exceeded 100% at 25 µg/ml and 50 µg/ml (Fig 3D). The kinetic profile showed that at these two higher concentrations, PLA_2_ activity remained above or equivalent to the positive control for the entire duration of the assay (Fig 3E and 3F). Notably, at 12.5 µg/ml, PLA_2_ activity was initially lower than the control but reached comparable levels after approximately 50 minutes. Full details on the protease and PLA_2_ activity assays are available in S4 and S5 Tables.

**Fig 4:**
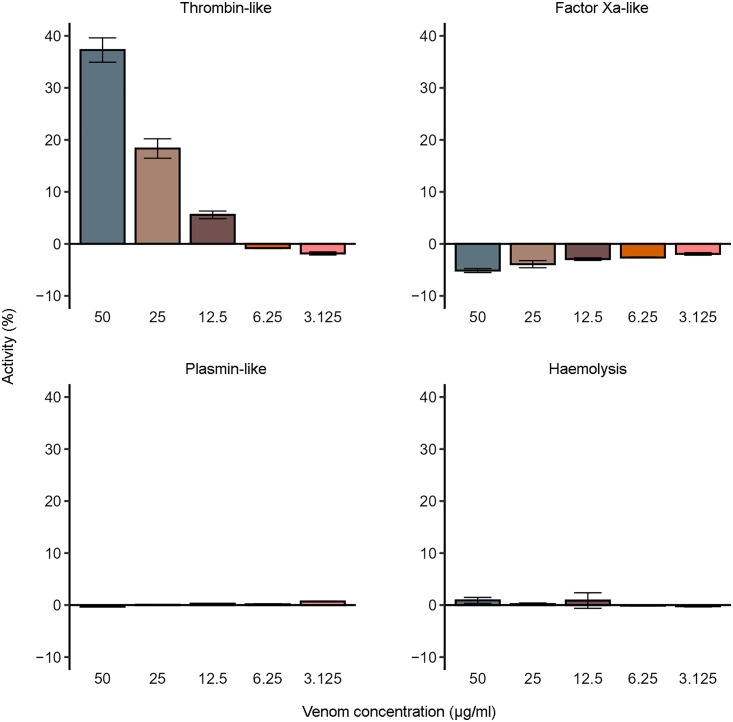
Biological activities of *E. coloratus* venom on different haematological targets. The values presented in each graph refer to the normalised mean values of three measurements per concentration. The error bars represent standard deviation. The positive controls used were Thrombin Enzyme Standard for the thrombin-like activity assay, FXa Enzyme Standard for the FXa-like activity assay, Plasmin Enzyme Standard for the plasmin-like activity assay, and Triton X-100 for the haemolysis assay. For all assays, ddH_2_O was used as the negative control.

In light of the reported haemostasis-altering effects of *E. coloratus* venom [28–30], we assessed its activity on several haematological targets. Thrombin-like activity showed a concentration-dependent increase, ranging from −1.8% (3.125 μg/ml) to 37.3% (50 μg/ml). In contrast, FXa-like, plasmin-like, and haemolytic activities were minimal across all tested concentrations, and no concentration-dependent patterns were evident. Particularly, FXa-like activity remained consistently negative, while the maximum recorded activities for plasmin-like and haemolytic activities were 0.9% (50 μg/ml) and 0.7% (3.125 μg/ml), respectively. The results of these assays are presented in Fig 4, and additional information is provided in S6–S9 Tables.

**Fig 5:**
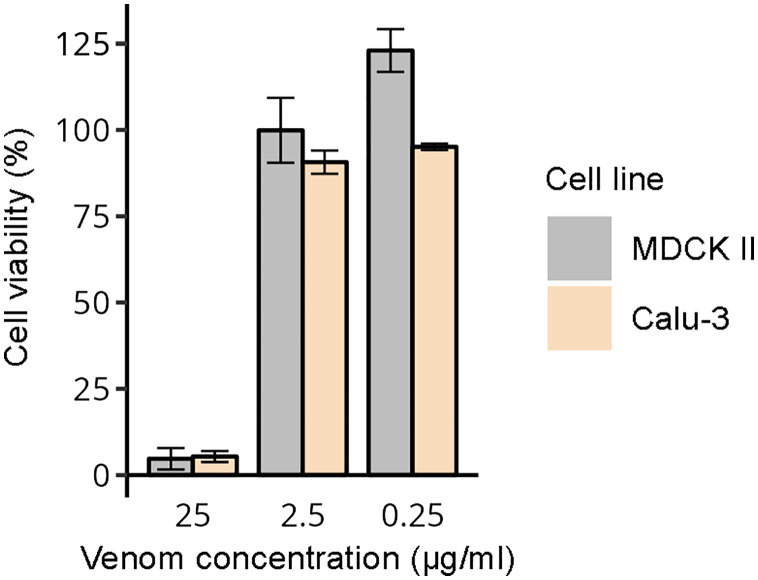
Cytotoxicity of *E. coloratus* venom. The graph shows the viability of the mammalian cell lines MDCK II and Calu-3 following treatment with the analysed venom. The error bars represent standard deviation.

Given previous reports of cytotoxicity associated with *E. coloratus* bites [27], we evaluated its effects on the mammalian cell lines MDCK II (canine kidney) and Calu-3 (human lung adenocarcinoma). At the highest venom concentration (i.e., 25 µg/ml), cytotoxicity reached 95.3% in MDCK II and 94.6% in Calu-3. In contrast, cytotoxic effects were minor at lower concentrations, with values below 10% at 2.5 µg/ml and 0.25 µg/ml in both cell lines (Fig 5). Further details on the cytotoxicity assays are available in S10 Table.

## Discussion

### Proteotranscriptomics of *Echis coloratus* venom points to intraspecific variation

In light of the medical importance of snakebite envenoming, elucidating the composition and biological activities of venoms from clinically relevant, yet understudied, snake species is of critical importance. Such investigations are essential for improving our understanding of envenomation pathophysiology and for supporting the development of more effective therapeutic strategies [3,5]. In this study, we present a comprehensive proteotranscriptomics-based characterisation of the diversity and activities of the toxin arsenal of the Arabian saw-scaled viper, *Echis coloratus*, a species of high medical relevance in North Africa and the Middle East, but one that has received comparatively limited attention in venom research.

To date, only one study by Casewell et al. [56] has employed a transcriptome-guided proteomic approach to investigate the venom composition of *E. coloratus*, which also remains the sole characterisation of this species’ toxin repertoire. Interestingly, the authors did not detect several toxin families we identified in the present work, such as VEGF, VNGF, and SVMPi/NP. Nevertheless, their findings revealed a venom composition broadly consistent with the general toxin profile observed in Old World vipers [20], with the principal toxin families SVMP, PLA_2_, CTL, and SVSP accounting collectively for 81% of venom components (53%, 13%, 11%, and 4%, respectively). Our analysis similarly indicates that these toxin families constitute 85% of the *E. coloratus* venom proteome (Fig 2C, S3 Table). However, our data show a marked predominance of CTL and PLA_2_, which together account for 73% of venom proteins, with CTLs alone contributing 44%. This composition differs from that reported by Casewell et al. [56] and commonly observed in the venom profiles of other *Echis* species, in which SVMPs are frequently the major venom components, sometimes representing up to 76% of total venom proteins [20,56,57]. In contrast, SVMPs were present at markedly lower levels in our study (5%; Fig 2C). Furthermore, these findings are corroborated by the results of the SDS-PAGE profiling, particularly under reducing conditions, where prominent bands are observed primarily between 13 and 15 kDa (Fig 2B). This molecular weight range corresponds to the estimated molecular masses of most CTLs and PLA_2_s previously identified in *E. coloratus* venom [56]. Given that CTLs are known to primarily disrupt haemostasis [58], and that PLA_2_s are associated with a broad spectrum of biological activities, including cytotoxicity and coagulopathy [59,60], the high relative abundances of these two toxin families in our data is consistent with the clinical symptoms documented in cases of envenomation by *E. coloratus* [27–31]. The HPLC venom profile displays a prominent signal (peak 2) corresponding to pEKW, featuring an N-terminal pyroglutamate residue (pE). This peptide was identified from two SVMPi/NP precursor sequences containing multiple QKW motifs (S2 Table). It is a well-characterised component of the venoms of many Old World vipers, in which it is often present at high abundance [20,61].

The integration of shotgun proteomics with species-specific omics-derived databases, such as venom gland transcriptomes, has been shown to provide a particularly comprehensive characterisation of venom diversity [62,63]. In this context, differences in terms of toxin diversity and abundances between our *E. coloratus* venom proteome and that presented by Casewell et al. may arise from the different methodologies applied (i.e., shotgun proteomics vs. “snake venomics”, respectively). Indeed, while these approaches can reveal similar general trends (e.g., in the most abundant toxin families), they are not directly interchangeable [64]. Nonetheless, the observed differences could also reflect unexplored venom variation within *E. coloratus*. In fact, although specimens from both studies originated from Egypt, local-scale variation in venom composition is well documented in vipers, and has been frequently linked to spatial heterogeneity in selective pressures [65–67]. Furthermore, it should be noted that while our study involved only adult individuals, the age of the specimens examined by Casewell et al. [56] was not specified. This leaves open the possibility that age-related venom variation, another common phenomenon in snakes [68–70], may have contributed to the observed differences.

### Functional profiling and clinical correlates of *E. coloratus* venom

Envenomation by *E. coloratus* can result in a broad spectrum of clinical manifestations, ranging from local effects such as necrosis at the bite site [27] to systemic haemotoxic symptoms, including haemorrhagic shock, thrombocytopenia, and coagulopathy [28–31]. Despite the recognised clinical relevance of *E. coloratus* venom, its biological activities have been scarcely investigated. In response to the lack of information on the functional features of *E. coloratus* venom, we conducted a comprehensive series of in vitro bioassays to characterise the functional profile of the Arabian saw-scaled viper’s venom.

Our assays focused on protease and PLA_2_ activities, two major enzymatic components of *E. coloratus* venom (Fig 2), revealed activities that were comparable to or exceeded those of the positive control, particularly at the three highest concentrations tested (i.e., 50, 25, and 12.5 µg/ml; Fig 3). Although proteases constitute only 12% of the *E. coloratus* venom proteome (Fig 2), the remarkable enzymatic activity observed suggests that, despite their relatively low abundance, these components are highly potent. A similar interpretation can be applied to PLA_2_s, which however make up a larger portion of the venom (28%; Fig 2), indicating that the elevated PLA_2_ activity detected may stem both from their high relative abundance and intrinsic potency. Interestingly, the kinetic profiles of both protease and PLA_2_ showed a rapid increase within the first 20 minutes of incubation. Proteases like SVMP and SVSP are among the major components of *Echis* venoms [20,56], and are among the principal drivers of haemotoxic effects [71], while PLA_2_s are frequently implicated in cytotoxicity [72,73]. In this context, our findings further underscore the functional significance of these enzymes in the venom of saw-scaled vipers.

Given the documented haemotoxic, primarily coagulopathic symptoms in *E. coloratus* envenomation cases [30,31], we investigated the venom’s Factor Xa-, thrombin-, and plasmin-like activities, as well as its haemolytic potential (Fig 4). Particularly, the Factor Xa-like and thrombin-like activity assays are designed to evaluate procoagulant enzymatic activities, as both Factor Xa and thrombin are key enzymes in the coagulation cascade responsible for promoting clot formation [74]. Conversely, the plasmin-like activity assay assesses fibrinolytic activity, which is associated with anticoagulant or clot-dissolving processes [75]. By including this assay, we aimed to detect any fibrinolytic components that may counterbalance clot formation and influence clinical outcomes, particularly in relation to consumptive coagulopathy. While we detected virtually no Factor Xa- or plasmin-like activities, the observed thrombin-like activity could promote the formation of unstable fibrin clots [76]. The repeated formation of unstable clots by thrombin-like snake venom serine proteases, commonly known as thrombin-like enzymes (TLEs), followed by their breakdown via plasmin, can lead to progressive fibrinogen depletion. This may ultimately result in TLE-mediated coagulopathy [77,78]. Nonetheless, it should be noted that *Echis* venoms are not typically associated with TLE-mediated coagulopathy, but rather with procoagulant venom-induced consumptive coagulopathy (VICC) mediated through the activation of coagulation factors [79].

Consistent with prior clinical reports of cytotoxicity following *E. coloratus* bites [27], the venom of this species exhibited pronounced cytotoxic effects at the highest concentration tested (i.e., 25 µg/ml) on the mammalian cell lines MDCK II and Calu-3 (Fig 5). The cytotoxic effects of viperid venoms are typically attributed to the action of proteases and PLA_2_s [72,73], which together account for approximately 40% of the *E. coloratus* venom proteome. Coupled with the strong enzymatic activities observed for these two venom component groups, our findings form a coherent narrative, strongly suggesting that proteases and PLA_2_s are likely to play a major role in determining the cytotoxicity and tissue damage reported in cases of *E. coloratus* envenomation.

## Conclusions

In the context of the global snakebite crisis and the limitations of current antivenom therapies, particularly in under-resourced regions of the world, a detailed understanding of the venom composition and functional effects of medically important, yet understudied, snake species is urgently required. To address this knowledge gap, we have presented a comprehensive proteotranscriptomics-based compositional and functional characterisation of the venom of the Arabian saw-scaled viper, *Echis coloratus*, one of the snake species of highest medical relevance in North Africa and the Middle East.

Our findings reveal a compositional profile that is notably distinct from those reported in other *Echis* species. Indeed, *E. coloratus* venom is characterised by an unusually high abundance of CTLs and PLA_2_s, alongside a striking scarcity of SVMPs, which represents a deviation from the SVMP-rich venoms typically observed in saw-scaled vipers. Despite the relatively low abundance of SVMPs and SVSPs, the venom displayed potent protease and PLA_2_ activities in vitro, and analysis of the kinetic profiles revealed rapid enzymatic activity within the first 20 minutes of incubation. Furthermore, pronounced cytotoxicity was observed at higher venom concentrations, consistent with clinical reports of tissue damage and necrosis at the bite site. While the thrombin-like activity observed may suggest the occurrence of TLE-mediated coagulopathy following envenomation by *E. coloratus*, the absence of FXa-like activity in this species is somewhat surprising, given that bites by saw-scaled vipers are typically known to induce procoagulant VICC via activation of coagulation factors. In light of these findings, future studies should aim to explore in greater detail the effects of *E. coloratus* venom, for example by investigating the mechanisms through which it influences coagulation factors and the complex interactions within plasma clotting cascades.

The divergence between our findings and the few, previous studies on *E. coloratus* venom may suggest the presence of intraspecific venom variability. While the underlying drivers of this variation remain unknown, their elucidation is of critical importance, particularly in the context of antivenom development. Indeed, antivenoms that fail to account for such variability may show reduced efficacy, thereby increasing the risk of poor clinical outcomes in envenomated patients.

Overall, this study represents arguably the most comprehensive investigation into the compositional and functional properties of *E. coloratus* venom available to date. Our findings underscore the importance of integrating detailed venom profiling with functional assays, and highlight the urgent need to investigate venom variability at the population level. Given the implications of intraspecific venom variation for both clinical outcomes and antivenom effectiveness, further investigation into venom variability within *E. coloratus* is essential for advancing our understanding of envenomation pathophysiology and for guiding the design and optimisation of more effective antivenoms.

## Supporting information

S1 Table Complete list of all components identified in the analysed *E. coloratus* venom. Reported are details concerning protein identification and annotation performed through PEAKS Studio 11.0. All venom-related (e.g., toxins typically found in snake venoms) and non-venom-related (e.g., physiological body proteins) components detected in the analysed venom are shown. Putative component IDs are assigned based on Description and database (i.e., UniProt, NCBI) search. The values in column “Venom-related” indicate whether a component is typically considered to play a role in envenomation or not.
(XLSX)

S2 Table List of the 183 reliably identified venom-related components. For each component, both Spectral Abundance Factor (SAF) and Normalized Spectral Abundance Factor (NSAF) are reported.
(XLSX)

S3 Table Cumulative Normalized Spectral Abundance Factor (NSAF) of each venom-related protein family reliably identified in the analysed *E. coloratus* venom. The NSAF values provide an estimate of the relative abundance of each protein family. The values are based on spectral counts, normalised by protein length and the total number of spectra in the analysed venom sample.
(XLSX)

S4 Table Raw data of the protease activity assay. TPCK trypsin was applied as positive control, and TBS as negative control.
(XLSX)

S5 Table Raw data of the phospholipase A_2_ activity assay. Bee venom PLA_2_ diluted in 1 × Reaction Buffer to a working solution of 10 U/ml PLA_2_ was applied as positive control, and 1 × Reaction Buffer as negative control.
(XLSX)

S6 Table Raw data of the Factor Xa-like activity assay. FXa Enzyme Standard was applied as positive control, and ddH_2_O as negative control.
(XLSX)

S7 Table Raw data of the thrombin-like activity assay. Thrombin Enzyme Standard was applied as positive control, and ddH_2_O as negative control.
(XLSX)

S8 Table Raw data of the plasmin-like activity assay. Plasmin Enzyme Standard was applied as positive control, and ddH_2_O as negative control.
(XLSX)

S9 Table Raw data of the haemolytic activity assay. The assay was performed on purified horse erythrocytes. Triton X-100 was applied as positive control, and ddH_2_O as negative control.
(XLSX)

S10 Table Raw data of the cytotoxicity assays performed on MDCK II and Calu-3 cells. Ionomycin was applied as positive control, and ddH_2_O as negative control.
(XLSX)
